# Perfusion Network Shift during Seizures in Medial Temporal Lobe Epilepsy

**DOI:** 10.1371/journal.pone.0053204

**Published:** 2013-01-14

**Authors:** Karen M. Sequeira, Ali Tabesh, Rup K. Sainju, Stacia M. DeSantis, Thomas Naselaris, Jane E. Joseph, Mark A. Ahlman, Kenneth M. Spicer, Steve S. Glazier, Jonathan C. Edwards, Leonardo Bonilha

**Affiliations:** 1 Comprehensive Epilepsy Center, Division of Neurology, Department of Neurosciences, Medical University of South Carolina, Charleston, South Carolina, United States of America; 2 Department of Radiology and Radiological Science, Medical University of South Carolina, Charleston, South Carolina, United States of America; 3 Division of Biostatistics and Epidemiology, Department of Medicine, Medical University of South Carolina, Charleston, South Carolina, United States of America; Emory University, Georgia Institute of Technology, United States of America

## Abstract

**Background:**

Medial temporal lobe epilepsy (MTLE) is associated with limbic atrophy involving the hippocampus, peri-hippocampal and extra-temporal structures. While MTLE is related to static structural limbic compromise, it is unknown whether the limbic system undergoes dynamic regional perfusion network alterations during seizures. In this study, we aimed to investigate state specific (i.e. ictal versus interictal) perfusional limbic networks in patients with MTLE.

**Methods:**

We studied clinical information and single photon emission computed tomography (SPECT) images obtained with intravenous infusion of the radioactive tracer Technetium- Tc 99 m Hexamethylpropyleneamine Oxime (Tc-99 m HMPAO) during ictal and interictal state confirmed by video-electroencephalography (VEEG) in 20 patients with unilateral MTLE (12 left and 8 right MTLE). Pair-wise voxel-based analyses were used to define global changes in tracer between states. Regional tracer uptake was calculated and state specific adjacency matrices were constructed based on regional correlation of uptake across subjects. Graph theoretical measures were applied to investigate global and regional state specific network reconfigurations.

**Results:**

A significant increase in tracer uptake was observed during the ictal state in the medial temporal region, cerebellum, thalamus, insula and putamen. From network analyses, we observed a relative decreased correlation between the epileptogenic temporal region and remaining cortex during the interictal state, followed by a surge of cross-correlated perfusion in epileptogenic temporal-limbic structures during a seizure, corresponding to local network integration.

**Conclusions:**

These results suggest that MTLE is associated with a state specific perfusion and possibly functional organization consisting of a surge of limbic cross-correlated tracer uptake during a seizure, with a relative disconnection of the epileptogenic temporal lobe in the interictal period. This pattern of state specific shift in metabolic networks in MTLE may improve the understanding of epileptogenesis and neuropsychological impairments associated with MTLE.

## Introduction

For most patients with medial temporal lobe epilepsy (MTLE), the hippocampus is presumed to be the location of seizure onset [1]. The most common histological finding in patients with MTLE is hippocampal cell loss and gliosis, which defines a well know pathological entity termed hippocampal sclerosis (HS) [2]. The successful removal of the affected hippocampus through neurosurgery can lead to seizure freedom in the majority of patients with MTLE [3].

Features of HS can be assessed in-vivo through the use of neuroimaging. In particular, structural imaging such as magnetic resonance imaging (MRI) can demonstrate abnormalities that are consistently associated with hippocampal sclerosis, such as hippocampal atrophy on T1 weighted images and increased hippocampal signal on T2 based sequences [4]. These abnormalities can be visually inspected on routine clinical scans, or quantified using post-processing techniques [5]. Fortunately, with recent improvements in image technology, the accuracy of determining the presence of hippocampal sclerosis is satisfactorily high when T1 or T2 hippocampal abnormalities can be observed or quantified [6].

For most patients with MTLE, structural neuroimaging alone is not sufficient to establish that seizures indeed arise from the hippocampus. In particular, for patients who do not achieve seizure control with medications and who are being considered for epilepsy surgery, it is of paramount importance to accurately identify whether the medial temporal region is the site of seizure onset. Even though the presence of signs associated with hippocampal sclerosis may suggest that the hippocampus is likely the epileptogenic area, features of HS have been observed in subjects without seizures and epilepsy [7]. Therefore, functional imaging is routinely performed in patients with MTLE who are undergoing surgical planning.

In general, once seizures have been confirmed to arise from the temporal lobe through video-electroencephalography (VEEG), functional neuroimaging techniques such as single photon emission computed tomography (SPECT) or positron emission tomography (PET) are used to provide evidence of medial temporal dysfunction and involvement during seizures. Since the spatial resolution of VEEG is suboptimal, combining evidence from techniques that can provide evidence of localized dysfunction such as structural MRI and SPECT or PET can increase the level of confidence of that the medial temporal region is the epileptogenic zone.

Among functional techniques, SPECT images can provide the unique contrast between interictal and ictal states, since SPECT images can be obtained during the ictal or interictal states, as confirmed by VEEG. The contrast state specific images can thus provide evidence of the metabolic ‘hot-spot’, where seizures are likely to arise or spread from. Importantly, SPECT is measure of perfusion changes, which are theoretically linked to the underlying neurophysiological ictal activity. It is an indirect measure and should be therefore interpreted with caution. Even though they cannot precisely confirm the primary neurophysiological process beyond the concurrent EEG, they offer the unique opportunity to evaluate a ‘within subject’ shift between the interictal and ictal state.

The use of quantifiable neuroimaging has recently provided significantly novel insight into the pathophysiology of MTLE. By enabling the in-vivo quantification of tissue abnormalities, structural MRI has disclosed that MTLE is a disease affecting not only the hippocampus, but also limbic structures that are anatomically or functionally related to the hippocampus [8]–[18]. Several recent studies have confirmed that tissue atrophy involves a network of limbic structures such as the medial temporal paleo and neocortex, insula, thalamus, cingulate regions and cerebellum, among others. Hence, the concept of MTLE as a network disease has gained popularity, and it is possible that a more accurate identification of previously ‘unseen’ abnormalities of network configuration may enable better a sub-categorization of MTLE, and may therefore serve as a biomarker to directly aid clinical decision.

Whole brain SPECT studies have confirmed structural imaging findings by demonstrating that there is a relative increase in radiotracer uptake during the ictal state in hippocampal, and extra-hippocampal regions, corroborating the notion of seizure spread. Quantifiable SPECT studies have demonstrated that this pattern is reproducible across patients and represents an in increase in metabolic demand within the limbic system [19], [20]. Furthermore, the increase in perfusion within epileptogenic structures can be associated with a correlated decrease in perfusion in other brains structures [20].

Nonetheless, it is largely unknown whether the functional organization of the limbic network is inherently abnormal between disease states. Is the limbic network configured in the same way in the ictal state compared to the interictal state, except for a higher radiotracer uptake? Or do seizures restructure the connectivity between structures in order to facilitate the flow of epileptogenic spread? Are regional abnormalities undisclosed by whole-brain SPECT subtraction analyses that may be revealed by ictal network analyses?

The motivation to investigate ictal changes as alterations of patterns of connectivity is derived from recent studies suggesting that network analyses may help elucidate the pathophysiology of epilepsy [21], [22]. Even though several studies have demonstrated regional abnormalities in volume, white matter connectivity and perfusion in MTLE, it is still unclear how these changes affect the conformation of the epileptogenic network in MTL [23].

In order to answer these questions, we evaluated state dependent (i.e., ictal versus interictal) pair-wise differences in SPECT images representing Technetium Tc-99 m Hexamethylpropyleneamine Oxime (Tc-99 m HMPAO) uptake in 20 patients with unilateral MTLE. We focused our analyses in the investigation of quantifiable measures of connectivity (via mathematical graph theory methods) of the whole network and specific limbic regions of interest (ROIs). We hypothesized that the ictal network demonstrates a significant functional reconfiguration of medial and posterior temporal regions.

## Methods

### Subjects

We studied 20 consecutive patients who were diagnosed with MTLE according to the parameters defined by the International League Against Epilepsy - ILAE [24]. All patients underwent a careful neurological assessment including neurological examination and video-electroencephalography monitoring. All patients had unilateral temporal seizure onset on VEEG. Eight patients had right MTLE and twelve had left MTLE. The mean age of patients was 36.7±14.8 years, and fifteen patients were women. The left MTLE group was similar to the right MTLE group in age (p = 0.68) and gender distribution (p = 0.19). The clinical and demographical details from the patient population evaluated in this study are summarized in Table 1. This study was approved by the IRB of the Medical University of South Carolina as a retrospective chart/imaging review without patient identifiers and without further steps beyond routine clinical care, under the category of ‘waiver of informed consent’.

**Table 1:** Table 1 demonstrates the clinical demographics for the patient population studied in this manuscript.

*Patient*	*Side*	*Age*	*Age of onset*	*Seizure frequency (szs per year)*	*SPECT injection time after seizure onset (seconds)*	*MRI findings*
1	Right	46	38	12	16	Right temporal cavernoma
2	Left	38	6	36	8	Normal
3	Right	9	5	24	5	Normal
4	Left	21	19	48	10	Left hippocampal T1 atrophy with increased hippocampal T2 signal.
5	Left	51	25	24	9	Left hippocampal T1 atrophy with increased hippocampal T2 signal.
6	Right	37	36	12	10	Normal
7	Right	30	10	96	8	Normal
8	Left	50	20	36	4	Left temporal non-contrast enhancing cyst
9	Left	59	1	12	11	Left hippocampal T1 atrophy with increased hippocampal T2 signal.
10	Left	37	23	36	12	Normal
11	Right	51	29	96	15	Right hippocampal atrophy
12	Right	13	11	48	10	Right hippocampal atrophy
13	Left	44	1	12	16	Normal
14	Left	24	6	48	13	Anterior left temporal pole encephalomalacia
15	Right	61	57	48	40	Normal
16	Right	23	18	144	4	Normal
17	Right	46	40	120	36	Normal
18	Left	35	30	24	14	Left hippocampal T1 atrophy with increased hippocampal T2 signal.
19	Left	41	2	24	32	Left hippocampal T1 atrophy with increased hippocampal T2 signal.
20	Left	19	2	96	15	Normal

### Imaging

All SPECT scans were performed at the Medical University of South Carolina. The ictal scans were performed when the patients were admitted to the Epilepsy Monitoring Unit, and seizures were confirmed by VEEG. The individual tracer injection times from the electrographic onset of the seizures are summarized in Table 1. All interictal scans were performed in the outpatient setting, commonly a few weeks after hospital discharge after patients had resumed their normal anti-epileptic and were at least 6 hours seizure free, per subjective report.

Ictal and interictal images were obtained after the intravenous infusion of the radioactive tracer Technetium Tc-99 m Hexamethylpropyleneamine Oxime (Tc-99 m HMPAO) and all patients were scanned in the same SPECT scanner (GE – Hawkeye).

Tomographic SPECT data was reconstructed into transverse slices without attenuation correction (IRNC format). All individual ictal and interictal SPECT images were normalized for whole brain radiotracer uptake. The cerebellum was used as the reference for relative uptake intensity using HERMES BRASS software suite. The images were then transferred in DICOM format onto a workstation where they were transformed into Nifti format using the software dcm2nii (http://www.mccauslandcenter.sc.edu/mricro/mricron/dcm2nii.html). The images were then non-linearly spatially normalized to a symmetrical SPECT template in standard space utilizing the normalization routines embedded in the software SPM8 (16 non-linear iterations) and the SPECT template provided with SPM8.

Left ictal and interictal images were left and right flipped along the y-axis, so that all images were in accordance regarding site of ictal onset. A voxel-wise paired t-test was applied to investigate differences between the ictal and interictal images between groups. Results were corrected for multiple comparisons utilizing a False Discovery Rate (FDR) [25] of q<0.05.

#### Connectivity matrices and graph theory measurements

Connectivity matrices were constructed for the ictal and interictal states through the following steps. The average voxel-wise radiotracer uptake was extracted from a modified version of the anatomical regions-of-interest (ROIs) defined by the intersection between the regions defined by the Anatomical Automatic Labeling atlas (http://www.cyceron.fr/web/aal__anatomical_automatic_labeling.html) [26] and SPECT images. The regions of interest and their respective stereotaxic coordinates are summarized in Table S1. In order to attenuate the effect of individual variability in regional tracer uptake, the average ROIs tracer uptake was normalized as a percentage of the global tracer uptake from the whole brain. These steps were performed using in-house developed MATLAB scripts and the software MRIcron (http://www.mccauslandcenter.sc.edu/mricro/mricron/).

A weighted non-directed connectivity matrix was then constructed for the interictal and ictal states, where each entry A_ij_ in the matrix ‘A’ represents the ‘link’ or connection strength between regions ‘i’ and ‘j’, and it corresponds the correlation coefficient R between the tracer uptake in regions ‘i’ and ‘j’.

In order to illustrate the absolute difference and percent of change between states, matrices illustrating the ‘Ictal-Interictal’ subtraction and ‘(Ictal/Interictal)-1’ percent of change were calculated.

The statistical differences between Ictal and Interictal connectivity matrices were then calculated used a permutation technique, whereby the labels (i.e., Ictal or Interictal) were permuted 10,000 times for each subject, for each region of interest. The comparison between the observed correlation coefficient between regions, and the distribution of correlation coefficients in the permuted data enabled the calculation of the probability of Type I error based on the observed correlation coefficient. A correction for multiple comparisons was performed by the “maximal statistic” method, which used for adjusting the p-values of each variable for multiple comparisons [27].

Graph theory-based measures were calculated employing the connectivity toolbox [28] (https://sites.google.com/a/brain-connectivity-toolbox.net/bct/). In order to overcome constraints in modern calculation regarding the presence of negative and positive ‘links’ in an adjacency matrix, graph measures were calculated only for positive links [29]. For each positive connectivity matrix (Inter and Interictal), we investigated measures related to 1) node properties, where each node corresponded to an anatomical ROI; and 2) global network properties.

The node properties evaluated in this study were 1) degree; 2) clustering coefficient, 3) local efficiency and 4) betweenness centrality. The percent of change in each node measure was then plotted anatomically employing the software BrainNet Viewer http://www.nitrc.org/projects/bnv/). The whole network measures applied in this study were 1) average degree, 2) average clustering coefficient, 3) global efficiency and 4) average betweenness centrality. The description of each measure and its significance within the context of network analysis is explained in Table S2.

In order to further explore the sampling distribution and potential changes between the Ictal and Interictal state, we also employed an exploratory bootstrapping strategy to evaluate the dispersion of the state-specific data, and ensuing graph measures.

The ROI data for each state (Ictal and Interictal) were submitted to independent sampling with replacement (bootstrapped) across subjects. For each ROI, bootstrapping was performed 10,000 times, and the resulting weighted non-directed connectivity matrix was calculated for each resampling. Labels (i.e., Ictal and Interictal) were not sampled; only the individual data entries for each ROI. From the resulting 20,000 matrices (10,000 Ictal and 10,000 Interictal), only the positive entries were maintained for subsequent graph theory measure. A series of fixed density binary matrices were then calculated for each repetition (10,000 Ictal and 10,000 Interictal matrices). The fixed density thresholds implied that on the sparsest matrix (density = 0.15), only the strongest 15% of all links were maintained. On the densest matrix, the strongest 45% links were maintained. The fixed density thresholds ranged from 0.05 to 0.45. The next step involved the calculation of whole network graph metrics for each binarized matrix: 1-) average degree, 2-) average clustering coefficient, 3-) global efficiency and 4-) average betweenness centrality. Importantly, given that each binarized fixed density threshold had a similar number of connections (highest 15%, etc), the average whole network degree was held constant for each threshold. Therefore, the calculation of average whole network was performed to ensure data and data processing quality.

The whole network measures were then submitted to statistical comparisons to evaluate for ictal versus interictal group differences. Assuming that bootstrapping involved an artificial generation of large samples, and therefore could strongly decrease the mean error of the sample, we decided to apply a very stringent correction for multiple comparisons. We employed a Bonferroni correction based on 1) the number of entries per each matrix (84*84); 2) the number of fixed density of thresholds (7); 3) the number of bootstrapped data (10,000 per group). The corrected p-value was therefore (0.05/[(84*84)*(7)*(20,000)]) = 5.0615e-11.

## Results

### Voxel-based comparison of tracer uptake

**Figure 1:**
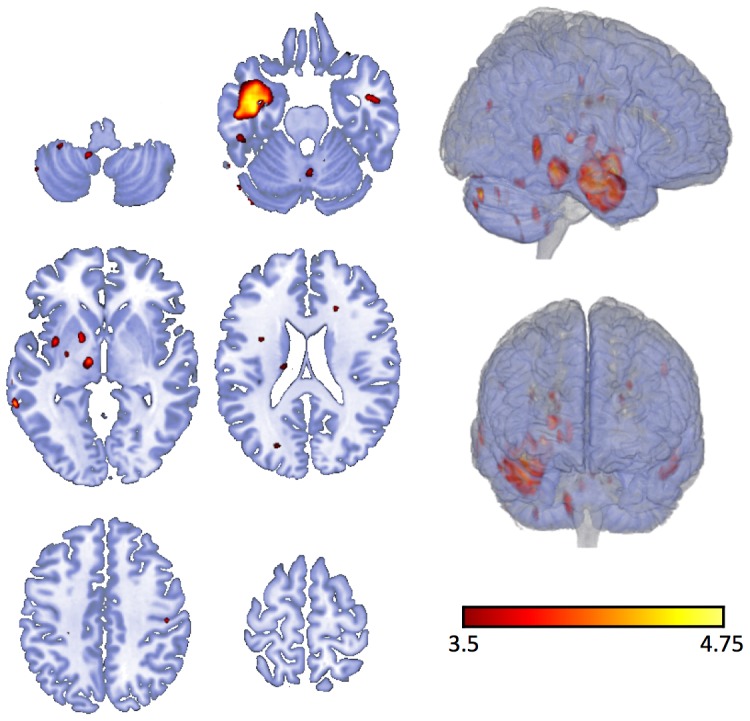
Regions with significantly higher uptake in Tc-99m HMPAO during the ictal state compared with the interictal state, based on paired sample t-test.

We observed a significant increase in Tc-99 m HMPAO uptake within the medial temporal lobe ipsilateral to the side of seizure onset, based on the voxel-wise paired t-test. A regional increase in tracer uptake was also observed in adjacent subcortical structures, notably the ipsilateral thalamus, pallidum, insula, cerebellum and white matter. Contralateral increase in tracer uptake was also in the medial temporal region, albeit to a lesser extent. There were no areas of significant decrement in tracer uptake during the ictal state. These results are summarized in Figure 1.

### Ictal and Interictal connectivity

**Figure 2:**
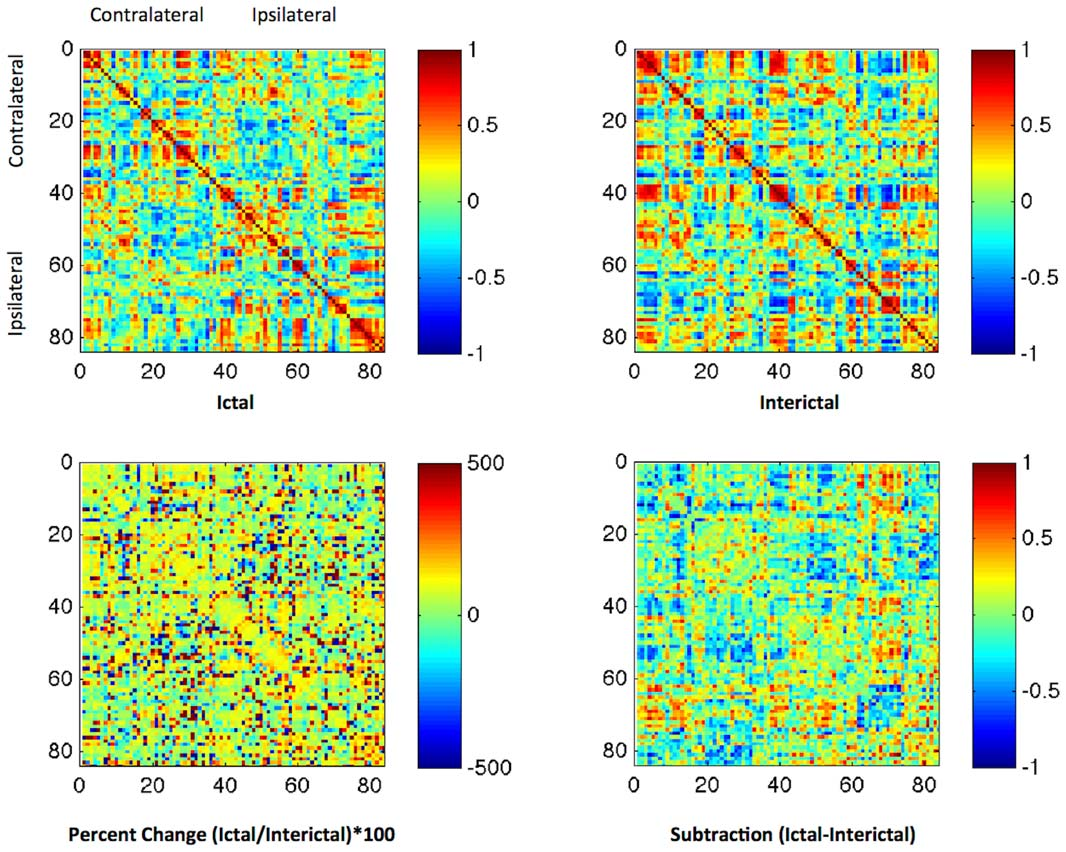
State dependent connectivity matrices. The upper panel displays the connectivity matrices illustrating the strength of connectivity across 84 regions of interest. The numbering of regions is equivalent to Table S1. Regions 1 to 42 represent the hemisphere contralateral to seizure onset, while 43 to 84 represent the homologous regions (1–43, 2–44, and so on) on the ipsilateral side. Each matrix entry represents the correlation coefficient “R” between each pair of regions. The lower panel demonstrates the percent change (ictal/interictal) and the difference between states.

The connectivity matrices from Ictal and Interictal states are demonstrated in Figure 2. Based on visual appreciation of the connectivity matrices, a notable decrement in global network connectivity could be appreciated in the Ictal matrix compared with the Interictal matrix. Higher correlation coefficients were more frequently observed in the Interictal state compared with the Ictal state. This contrast is highlighted by the relative percent change and difference between matrices also demonstrated in Figure 2. In general, the relative decrement of connectivity could be noted across multiple regions within the matrix, except for the medial temporal region, were the frequency of higher correlation coefficients was increased in the Ictal state.

**Figure 3:**
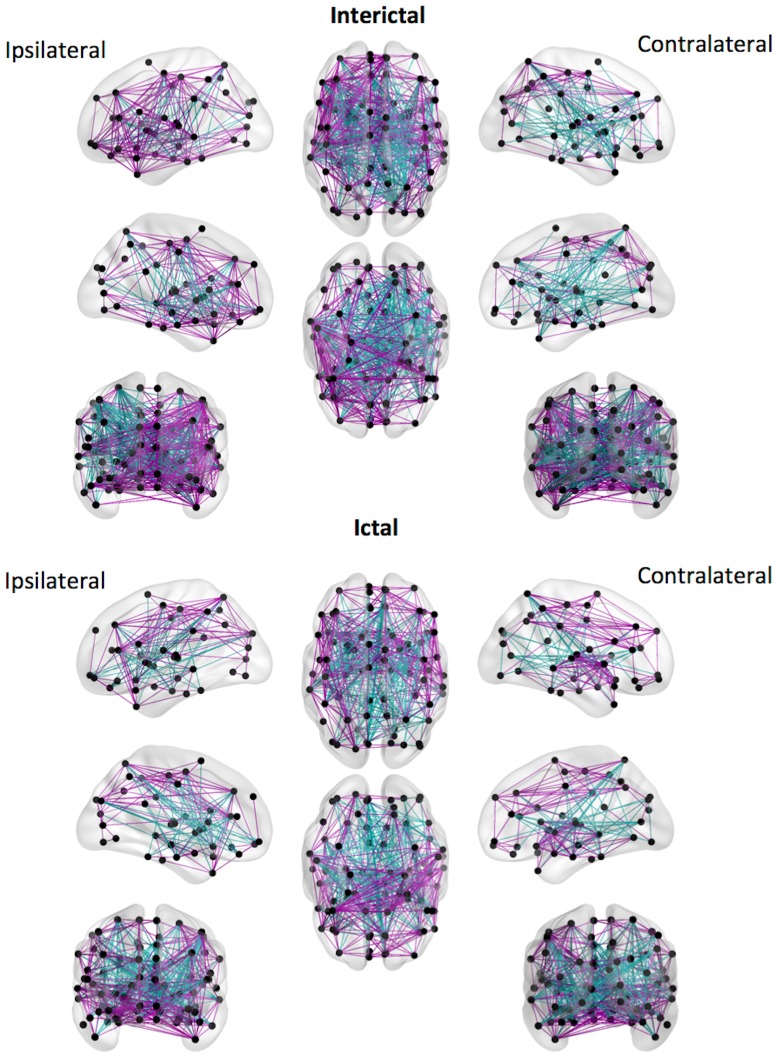
The anatomical distribution of positive (purple) and negative (cyan) links during the ictal and interictal states. A change in the number and distribution of positive and negative links can be observed in the ictal state compared with the interictal state. In the ictal state, a higher number of positive links is observed over the ipsilateral temporal lobe, with a concurrent reduction of positive links in extra-temporal regions.

To better illustrate this difference, Figure 3 demonstrates the overall distribution of regional links based on positive and negative correlation coefficients plotted over a diagram representing the brain and the stereotaxic centers of mass for the ROIs. During the Interictal state a substantial number of positive correlations can be seen diffusely overlying both hemispheres, with a relative decrement over the ipsilateral temporal lobe, possibly suggesting a regional cortical dysfunction of the epileptogenic region. This is replaced a strikingly different pattern during the interictal state, where a significant increase in connectivity is appreciated over the ipsilateral medial temporal region. Interestingly, a global decrement in connectivity is observed over most of the remaining brain network, suggesting a remarkable ‘down-state’ of the non-epileptogenic networks during a seizure.

**Figure 4:**
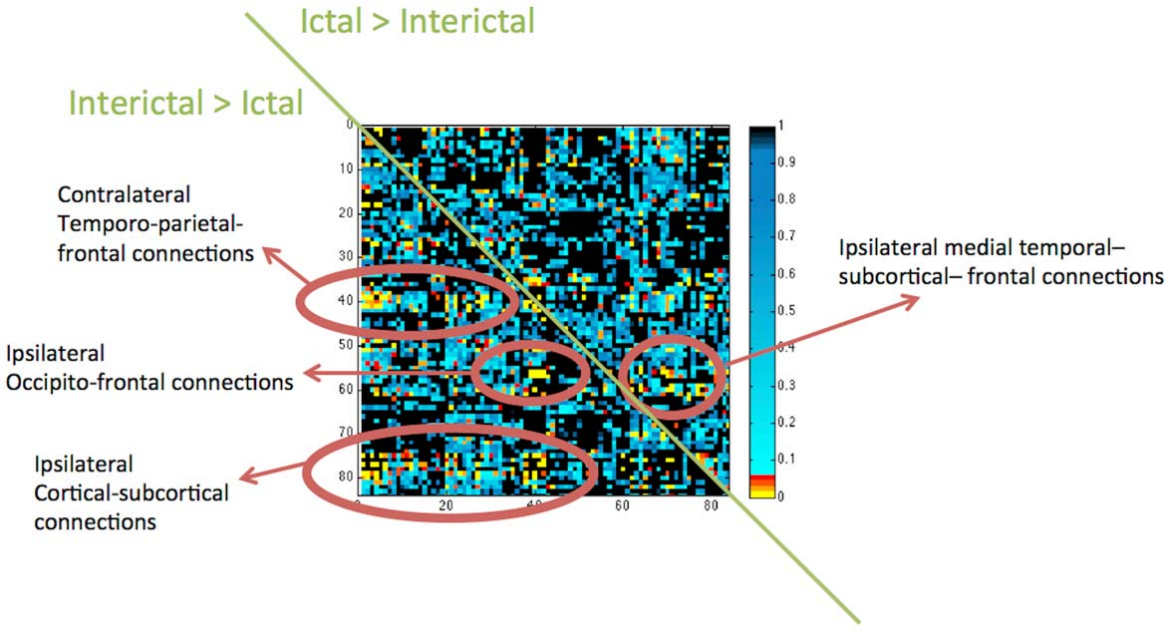
Statistical differences between the ictal and interictal state. These differences are displayed in a matrix of p-values. The row and column distribution is equivalent to the connectivity matrices displayed in previous figures (x-axis and y-axis ranging from regions 1 to 84). However, the upper triangular matrix represents the location were there was a significant ictal>interictal connectivity, while the lower triangular matrix represents interictal>ictal. These results are corrected for significant comparisons. The figure also highlights clusters of significant links with a legend to explain the corresponding anatomical location.

A more rigorous statistical analysis of the difference between the ictal and interictal states was performed by permutation tests, and the results can be seen in Figure 4. In general, the ictal state represents a significant increase in connectivity within the ipsilateral medial, temporal, subcortical, and frontal connections. Conversely, during the Interictal state, higher connectivity can be appreciated over the contralateral temporo-parietal-frontal connections, ipsilateral occipito-frontal connections and ipsilateral cortical-subcortical connections.

### Graph metrics

During the ictal state, noticeable changes in nodal graph measures were appreciated. There was a significant increase in nodal strength within the ipsilateral medial temporal region and adjacent subcortical structures. Nodal strength represents the multiplication of the number of connections per node by their strength, therefore indicating that connections are significantly ‘drawn’ to the ipsilateral temporal lobe and subcortical limbic structures. Conversely, a decrement in strength simultaneously occurs in distant brain areas during a seizure, more noticeably over the contralateral hemisphere, suggesting that these connections are temporarily ‘shut-down’ or attenuated during a seizure.

Local nodal efficiency was also increased in the ipsilateral temporal region, and medial subcortical regions, with a relative decrement in contralateral neocortical structures. Local efficiency is inversely related to regional path length, suggesting that the connectivity distance between medial temporal regions is increased during a seizure, while concurrent decrease is observed in contralateral and neocortical structures.

The nodal clustering coefficient demonstrated a very similar pattern of ictal related changes compared with local efficiency. The two metrics are inter-related, with the clustering coefficient indicating the number of ‘triangular’ connections to a node, largely representing reentrant connections.

Finally, a large shift in betweenness centrality was observed between states, with multiple temporal and subcortical regions displaying an increase in centrality, with widespread decrement in centrality over cortical regions in the brain convexity. Between centrality is a measure of how much a node is related to network shortest paths, therefore representing how ‘close’ the node is to the main connectivity pathway, or how ‘central’ the node is to the main connections. These results indicate a significant functional network reconfiguration during a seizure due to a shift in the main component of the network to subcortical and medial regions, with distancing of the cortical convexity structures.

**Figure 5:**
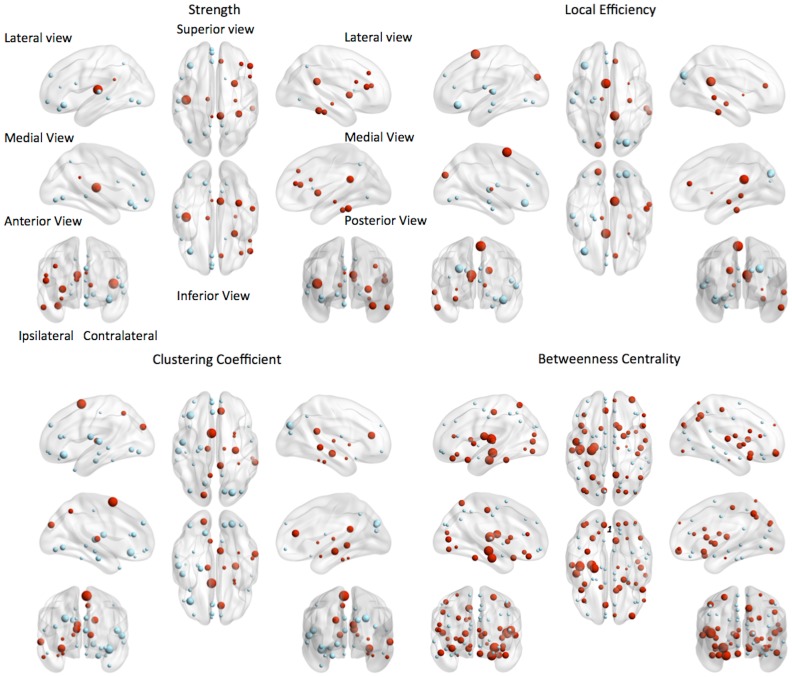
Changes in nodal graph properties in the ictal state compared with the interictal state. For all measures, nodes demonstrating at least 25% of change between states are shown. The nodes are displayed according to their stereotaxic location, and red represents a change ictal>interictal, and blue interictal>ictal. The size of the node is relatively proportional to the size of positive changes.

Importantly, the node metrics described above were calculated on positive connectivity matrices to enable the conventional calculation of graph properties. This may indicate that negative shifts in connectivity can be underestimated. Nodal graph results are summarized in Figure 5.

**Figure 6:**
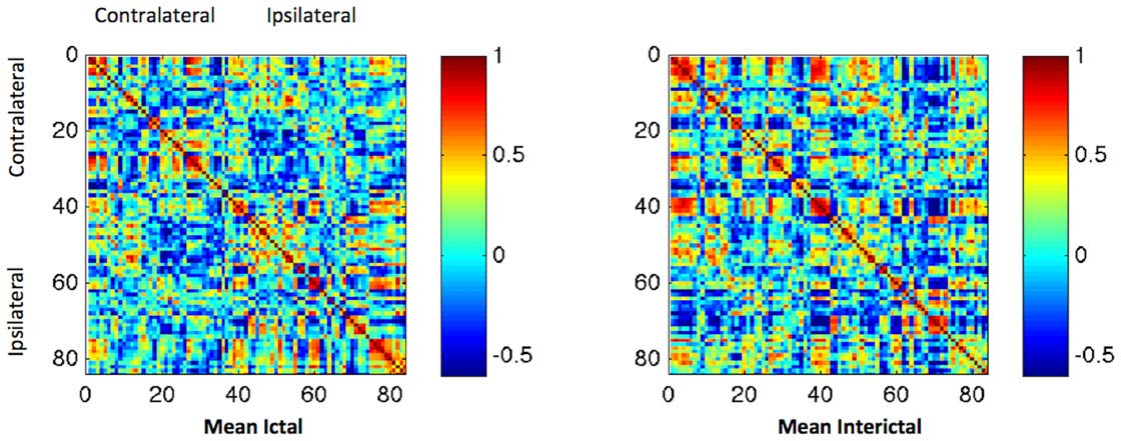
The average connectivity between pairs of regions, generated by bootstrapping the regional tracer uptake in each individual 10,000 times.

**Figure 7:**
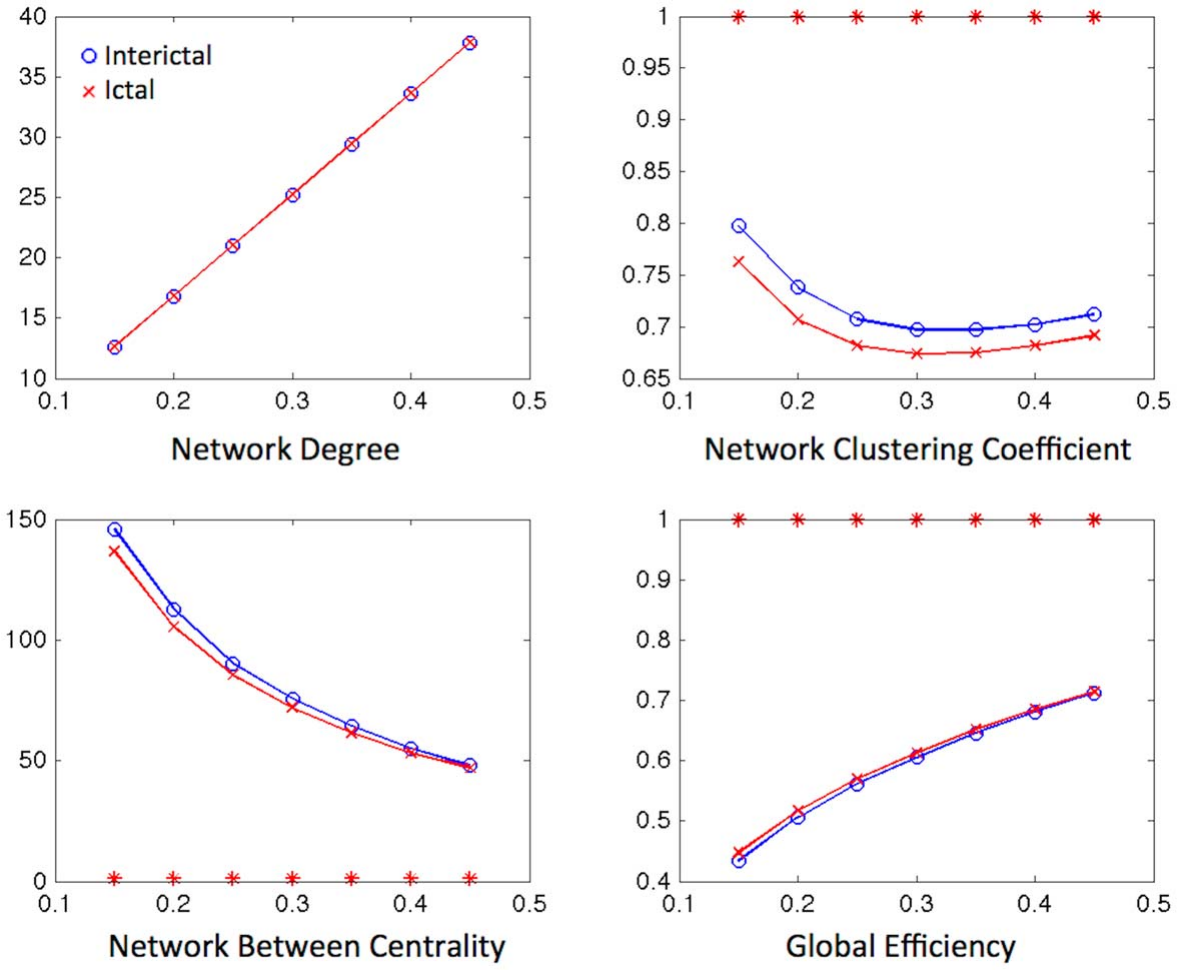
Average global network metrics. The global network metrics (y-axis) from the bootstrapped data are displayed across fixed density thresholds (x-axis) for binarized matrices. Statistically significant differences are highlighted with a red star.

Lastly, the data was bootstrapped and used to construct a series of binarized matrices based on density thresholds. This data was used to evaluate whole graph network measures. Interestingly, this analysis may overcome the limitations of the previous nodal calculations, as it may enable a better appreciation of negative shifts in connectivity, which may become apparent as a regional decrement in connectivity at higher density thresholds. Figure 6 displays the averages of bootstrapped matrices for each state. Global network metrics are displayed in Figure 7. As expected, even though nodal metrics indicate an increase in connectivity within medial temporal and subcortical structures during the Ictal state, there is an overall decrement in normal segregation, accompanied by increased regional integration possibly due to vastly increased limbic connectivity. This indicates that the ictal state is associated with a regional hyperconnectivity, in addition to a relative attenuation of global connectivity.

## Discussion

In this manuscript, we investigated state-specific changes in functional connections in patients with MTLE. By examining regional uptake of Tc-99 m HMPAO during the ictal and interictal states, we constructed a pattern of connectivity based on the correlation coefficients of brain regions for each state. Graph measures were then calculated to evaluate changes in network configuration that may not be apparent by exclusively qualitative visual analysis of images of the scans or the network.

The main findings from this study can be summarized as follows. First, as demonstrated by the voxel-based analysis, there is a relative increase in tracer uptake within the medial temporal region, lateral temporal cortex and the immediately adjacent subcortical and limbic regions. This is an intuitive observation, as this pattern is often observed during the pre-surgical analysis of individual patients. These findings have been reported before with similar analyses [19], [20], and they corroborate that our data fits the expected pattern of physiological ictal versus interictal perfusion. Importantly, these findings alone cannot elucidate changes in the global network configuration because little information can be obtained about the influence of each brain region, in particular those without significant voxel-based perfusion changes, to the global brain network.

The subsequent analyses relied on the construction of connectivity matrices and the calculation of graph measures from state dependent networks. SPECT data represents a measure of blood flow, which is linked to metabolism, which is in turn linked to the underlying metabolic demand driven by neurophysiology activity. This is the premise underlying the clinical use of SPECT, since SPECT can reliably suggest the location of ictal onset or ictal spread through changes in metabolism [30]–[33]. It is important to note that there is an expected time lag between injection and radiotracer uptake, which usually ranges in the order of 30 seconds with individual variability [34]. Therefore, the SPECT data represent a pattern of blood flow usually seconds after the seizure onset, which is associated with the metabolic demand driven by underlying neurophysiology. The analyses of networks infer that regions which show a correlated change in SPECT tracer uptake between states are connected by exhibiting a similar change in metabolic demand which is turn related to a similar driving neurophysiologic changes. This is should be kept in perspective when interpreting the following results. Similarly, the term functional connectivity is employed assuming that correlated regions may be indirectly connected by similar changes in metabolic demand, but it should also be interpreted in this context.

The second analysis consisted of constructing the connectivity matrices and plotting the pattern of connectivity on anatomical templates, we observed that the interictal state is associated with a pattern of widespread positive correlations between cortical and subcortical structures. This pattern is attenuated over the ipsilateral hemisphere. Theoretically, this may represent a normal pattern of brain connectivity, and its disruption over the ipsilateral medial temporal lobe likely represents the hypo-functionality of the epileptogenic region. These observations are in accordance with a model for MLTE supporting a functional impairment of the ipsilateral hippocampus and medial temporal lobe with concurrent compensatory function from the contralateral hemisphere [35].

Importantly, during the ictal state, a global change in the network organization was observed. There was an increased correlation of tracer uptake within medial temporal and limbic regions, and a global decrease in cross-correlated tracer uptake throughout the remaining cortex. The network formed by correlated areas in the interictal state was replaced by a very different ictal network, with relative suppression of the remaining cortex, and increased connectivity of the medial temporal region. Interestingly, during the interictal state, the medial temporal region showed a relative disconnection with the remaining network. This observation corroborates the current concept that pathological epileptogenic regions in MTLE are not cognitively functional, but can experience a surge of activity during a seizure, thus the surgical removal of the epileptogenic area may not yield cognitive deficits and may actually be associated with a clinical cognitive improvement [36].

Third, through graph nodal network analyses, we observed that nodes within the medial temporal lobe exert a strong influence over the network integration due to their increase in local efficiency and betweenness centrality, while maintaining a high segregation pattern defined by a regional high clustering coefficient and strength. Conversely, both integration and segregation measures are strongly diminished over diffuse non-epileptogenic cortical and subcortical regions. These findings further illustrate a significant shift in network configuration due to a strong state-dependent change in the roles of brain regions. The epileptogenic and adjacent structures assume a more focal and centralized Ictal network, as previously demonstrated with structural brain MRI data [21].

Fourth and finally, by analyzing global network properties through a series of binarized matrices from bootstrapped data, we observed that the ictal state is associated with global decrement in betweenness centrality and clustering coefficient, with a concurrent increase in global efficiency. These findings may indicate that even though there is a regional increase in clustering coefficient and betweenness centrality in epileptogenic regions, the overall result of the ictal state in the global network is a general decrease in its average clustering coefficient and betweenness centrality due a decrease in these measures in the many nodes in the network. Interestingly, the increase in local nodal efficiency during the ictal state yields higher global efficiency during the ictal state. Importantly, these results do not signify that the global efficiency is higher in epilepsy in general, but it indicates that there is a shift in global network properties in between states. Interestingly, previous neurophysiological studies have demonstrated somewhat conflicting results, possibly due to different methodologies. For example, Schindler et al. investigated global network changes derived from scalp EEG data and observed that seizures are associated with a transient shift towards a more regular network topology [37]. They observed that during a seizure, the global network clustering coefficient was increased compared to the pre-seizure state and compared with a random network. In this study, we observed a decrement in global clustering coefficient, even though the ictal zone displays a large increment in clustering coefficient during a seizure. This apparent discrepancy may therefore be a reflection of the better spatial resolution of SPECT, or the increased sensitivity of scalp EEG to the ictal onset. Other interesting studies by Kramer et al demonstrated that path length and the betweenness centralization increase at seizure onset with no significant changes in clustering coefficient [38], and network synchronization increases at the onset but not during a seizure [39]. However, it is very important to note that these study investigated data from intracranial recordings including mostly patients with extra-temporal epilepsy, therefore likely representing much more localized view of ictal activity (i.e., restricted to the coverage of intracranial electrodes) and possibly a different pathological mechanism since not all seizures had limbic onset. Similarly, Ortega et al observed that the lateral neocortical temporal region exhibit highly differentiated patterns on clustering during seizures in patients with MTLE, and similar to previously mentioned studies evaluating data from intracranial studies, the data analyzed only involved a localized portion of the lateral neocortex [40].

Overall, our results indicate that MTLE is associated with significant shifts in functional network properties. This information cannot be perceived by visual inspection or voxel-wise analyses alone, but indicates a reconfiguration of the network, notably through a breakdown of diffuse brain connectivity accompanied by a surge of connectivity within the epileptogenic regions. These findings support the concept that the epileptogenic region in MTLE is associated with paroxysmal waves of hyperconnectivity, but they also illustrate how the remaining network is also intermittently attenuated. It can be therefore hypothesized that frequent changes in network reconfiguration, notably through a breakdown of the normal pattern and its replacement by the ictal pattern, may lead to abnormal brain plasticity and long term reconfiguration towards a more epileptogenic and less cognitively efficient network. The frequent state transition in MTLE may lead to a chronic network disruption in MTLE, and the quantification of the chronic network abnormality may improve the understanding of epileptogenesis and treatment response.

## Supporting Information

Table S1 enumerates the regions of interest utilized in this study, with their respective stereotaxic coordinate.
(DOC)

Table S2 describes the global network and regional graph theory metrics employed in this study.
(DOC)
