# Supplementary material for: Perfusion Network Shift during Seizures in Medial Temporal Lobe Epilepsy
Source: PLoS One. 2013 Jan 14;8(1):e53204. doi: 10.1371/journal.pone.0053204 (PMC3544909; doi:10.1371/journal.pone.0053204)
Supplement: Table S1 — enumerates the regions of interest utilized in this study, with their respective stereotaxic coordinate. (DOC) [file pone.0053204.s001.doc]

**Supplementary Table S1** enumerates the regions of interest utilized in this study, with their respective stereotaxic coordinate.

| ***Side*** | ***Region Number*** | ***Region*** | ***X*** | ***Y*** | ***Z*** | ***Side*** | ***Region Number*** | ***Region*** | ***X*** | ***Y*** | ***Z*** |
| --- | --- | --- | --- | --- | --- | --- | --- | --- | --- | --- | --- |
| Contralateral | 1 | ***Precentral gyrus*** | -41.37 | -8.21 | 52.09 | Ipsilateral | 43 | ***Precentral gyrus*** | 41.37 | -8.21 | 52.09 |
| Contralateral | 2 | ***Middle frontal gyrus*** | -33.43 | 32.73 | 35.46 | Ipsilateral | 44 | ***Middle frontal gyrus*** | 37.59 | 33.06 | 34.04 |
| Contralateral | 3 | ***Middle orbitofrontal region*** | -30.65 | 50.43 | -9.62 | Ipsilateral | 45 | ***Middle orbitofrontal region*** | 33.18 | 52.59 | -10.73 |
| Contralateral | 4 | ***Inferio frontal gyrus (Opercular)*** | -48.43 | 12.73 | 19.02 | Ipsilateral | 46 | ***Inferio frontal gyrus (Opercular)*** | 50.2 | 14.98 | 21.41 |
| Contralateral | 5 | ***Inferio frontal gyrus (Triangular)*** | -45.58 | 29.91 | 13.99 | Ipsilateral | 47 | ***Inferio frontal gyrus (Triangular)*** | 50.33 | 30.16 | 14.17 |
| Contralateral | 6 | ***Inferior orbitofrontal region*** | -35.98 | 30.71 | -12.11 | Ipsilateral | 48 | ***Inferior orbitofrontal region*** | 41.22 | 32.23 | -11.91 |
| Contralateral | 7 | ***Rolandic region*** | -47.16 | -8.48 | 13.95 | Ipsilateral | 49 | ***Rolandic region*** | 52.65 | -6.25 | 14.63 |
| Contralateral | 8 | ***Supplementary motor area*** | -5.32 | 4.85 | 61.38 | Ipsilateral | 50 | ***Supplementary motor area*** | 8.62 | 0.17 | 61.85 |
| Contralateral | 9 | ***Orbital lateral frontal region*** | -8.06 | 15.05 | -11.46 | Ipsilateral | 51 | ***Orbital lateral frontal region*** | 10.43 | 15.91 | -11.26 |
| Contralateral | 10 | ***Medial superior frontal gyrus*** | -4.8 | 49.17 | 30.89 | Ipsilateral | 52 | ***Medial superior frontal gyrus*** | 9.1 | 50.84 | 30.22 |
| Contralateral | 11 | ***Medial superior orbital frontal region*** | -5.17 | 54.06 | -7.4 | Ipsilateral | 53 | ***Medial superior orbital frontal region*** | 8.16 | 51.67 | -7.13 |
| Contralateral | 12 | ***Rectus gyrus*** | -5.08 | 37.07 | -18.14 | Ipsilateral | 54 | ***Rectus gyrus*** | 8.35 | 35.64 | -18.04 |
| Contralateral | 13 | ***Insula*** | -35.13 | 6.65 | 3.44 | Ipsilateral | 55 | ***Insula*** | 39.02 | 6.25 | 2.08 |
| Contralateral | 14 | ***Anterior cingulate gyrus*** | -4.04 | 35.4 | 13.95 | Ipsilateral | 56 | ***Anterior cingulate gyrus*** | 8.46 | 37.01 | 15.84 |
| Contralateral | 15 | ***Dorsal cingulate gyrus*** | -5.48 | -14.92 | 41.57 | Ipsilateral | 57 | ***Dorsal cingulate gyrus*** | 8.02 | -8.83 | 39.79 |
| Contralateral | 16 | ***Posterior congulate gyrus*** | -4.85 | -42.92 | 24.67 | Ipsilateral | 58 | ***Posterior congulate gyrus*** | 7.44 | -41.81 | 21.87 |
| Contralateral | 17 | ***Hippocampus*** | -25.03 | -20.74 | -10.13 | Ipsilateral | 59 | ***Hippocampus*** | 29.23 | -19.78 | -10.33 |
| Contralateral | 18 | ***Parahippocampal gyrus*** | -21.17 | -15.95 | -20.7 | Ipsilateral | 60 | ***Parahippocampal gyrus*** | 25.38 | -15.15 | -20.47 |
| Contralateral | 19 | ***Amygdala*** | -23.27 | -0.67 | -17.14 | Ipsilateral | 61 | ***Amygdala*** | 27.32 | 0.64 | -17.5 |
| Contralateral | 20 | ***Calcarine fissure*** | -7.14 | -78.67 | 6.44 | Ipsilateral | 62 | ***Calcarine fissure*** | 15.99 | -73.15 | 9.4 |
| Contralateral | 21 | ***Cuneus*** | -5.93 | -80.13 | 27.22 | Ipsilateral | 63 | ***Cuneus*** | 13.51 | -79.36 | 28.23 |
| Contralateral | 22 | ***Lingual gyrus*** | -14.62 | -67.56 | -4.63 | Ipsilateral | 64 | ***Lingual gyrus*** | 16.29 | -66.93 | -3.87 |
| Contralateral | 23 | ***Suprerio occipital region*** | -16.54 | -84.26 | 28.17 | Ipsilateral | 65 | ***Suprerio occipital region*** | 24.29 | -80.85 | 30.59 |
| Contralateral | 24 | ***Middle occipital region*** | -32.39 | -80.73 | 16.11 | Ipsilateral | 66 | ***Middle occipital region*** | 37.39 | -79.7 | 19.42 |
| Contralateral | 25 | ***Inferior occipital region*** | -36.36 | -78.29 | -7.84 | Ipsilateral | 67 | ***Inferior occipital region*** | 38.16 | -81.99 | -7.61 |
| Contralateral | 26 | ***Fusiform gyrus*** | -31.16 | -40.3 | -20.23 | Ipsilateral | 68 | ***Fusiform gyrus*** | 33.97 | -39.1 | -20.18 |
| Contralateral | 27 | ***Postcentral gyrus*** | -42.46 | -22.63 | 48.92 | Ipsilateral | 69 | ***Postcentral gyrus*** | 41.43 | -25.49 | 52.55 |
| Contralateral | 28 | ***Superior parietal region*** | -23.45 | -59.56 | 58.96 | Ipsilateral | 70 | ***Superior parietal region*** | 26.11 | -59.18 | 62.06 |
| Contralateral | 29 | ***Inferior parietal region*** | -42.8 | -45.82 | 46.74 | Ipsilateral | 71 | ***Inferior parietal region*** | 46.46 | -46.29 | 49.54 |
| Contralateral | 30 | ***Supramarginal gyrus*** | -55.79 | -33.64 | 30.45 | Ipsilateral | 72 | ***Supramarginal gyrus*** | 57.61 | -31.5 | 34.48 |
| Contralateral | 31 | ***Angular gyrus*** | -44.14 | -60.82 | 35.59 | Ipsilateral | 73 | ***Angular gyrus*** | 45.51 | -59.98 | 38.63 |
| Contralateral | 32 | ***Precuneus*** | -7.24 | -56.07 | 48.01 | Ipsilateral | 74 | ***Precuneus*** | 9.98 | -56.05 | 43.77 |
| Contralateral | 33 | ***Caudate*** | -11.46 | 11 | 9.24 | Ipsilateral | 75 | ***Caudate*** | 14.84 | 12.07 | 9.42 |
| Contralateral | 34 | ***Putamen*** | -23.91 | 3.86 | 2.4 | Ipsilateral | 76 | ***Putamen*** | 27.78 | 4.91 | 2.46 |
| Contralateral | 35 | ***Pallidum*** | -17.75 | -0.03 | 0.21 | Ipsilateral | 77 | ***Pallidum*** | 21.2 | 0.18 | 0.23 |
| Contralateral | 36 | ***Thalamus*** | -10.85 | -17.56 | 7.98 | Ipsilateral | 78 | ***Thalamus*** | 13 | -17.55 | 8.09 |
| Contralateral | 37 | ***Heschl gyrus*** | -41.99 | -18.88 | 9.98 | Ipsilateral | 79 | ***Heschl gyrus*** | 45.86 | -17.15 | 10.41 |
| Contralateral | 38 | ***Superior temporal gyrus*** | -53.16 | -20.68 | 7.13 | Ipsilateral | 80 | ***Superior temporal gyrus*** | 58.15 | -21.78 | 6.8 |
| Contralateral | 39 | ***Superior temporal parietal occipital junction*** | -39.88 | 15.14 | -20.18 | Ipsilateral | 81 | ***Superior temporal parietal occipital junction*** | 48.25 | 14.75 | -16.86 |
| Contralateral | 40 | ***Middle temporal gyrus*** | -55.52 | -33.8 | -2.2 | Ipsilateral | 82 | ***Middle temporal gyrus*** | 57.47 | -37.23 | -1.47 |
| Contralateral | 41 | ***Middle temporal parietal occipital junction*** | -36.32 | 14.59 | -34.08 | Ipsilateral | 83 | ***Middle temporal parietal occipital junction*** | 44.22 | 14.55 | -32.23 |
| Contralateral | 42 | ***Inferior temporal gyrus*** | -49.77 | -28.05 | -23.17 | Ipsilateral | 84 | ***Inferior temporal gyrus*** | 53.69 | -31.07 | -22.32 |
