# Supplementary material for: Perfusion Network Shift during Seizures in Medial Temporal Lobe Epilepsy
Source: PLoS One. 2013 Jan 14;8(1):e53204. doi: 10.1371/journal.pone.0053204 (PMC3544909; doi:10.1371/journal.pone.0053204)
Supplement: Table S2 — describes the global network and regional graph theory metrics employed in this study. (DOC) [file pone.0053204.s002.doc]

**Supplementary Table S2** describes the global network and regional graph theory metrics employed in this study.

| **Graph theoretical property** | **Explanation** |
| --- | --- |
| *Node measures:* | |
| Nodal degree | Number of links connected to a node. |
| Clustering coefficient | Represents the fraction of a given node’s neighbors that are neighbors of each other. |
| Local efficiency | Average of the inverse shortest path length in the neighborhood of the node. |
| Betweenness Centrality | Represents the fraction of all shortest paths in the network that contain a given node. |
| *Global Network measurements:* | |
| Average degree | The average degree from all nodes in the network. |
| Average clustering coefficient | The average clustering coefficient from all nodes in the network. |
| Global efficiency | The average inverse shortest path length in the network |
| Average Betweenness Centrality | The average betweenness centrality from all nodes in the network. |
